# Oral Sex May Serve as Low Mate Value Compensation Among Men: Evidence from a Pre-registered Study

**DOI:** 10.1007/s10508-024-03064-4

**Published:** 2024-12-26

**Authors:** Natalia Frankowska, Aleksandra Szymkow, Andrzej Galbarczyk

**Affiliations:** 1 Center for Research on Biological Basis of Social Behavior, SWPS University, Warsaw, Poland; 2 Department of Human Behavior, Ecology and Culture, Max Planck Institute for Evolutionary Anthropology, Deutscher Platz 6, 04103 Leipzig, Germany; 3 Department of Environmental Health, Faculty of Health Sciences, Jagiellonian University Medical College, Krakow, Poland

**Keywords:** Oral sex, Cunnilingus, Mate value, Sexual behavior, Mate retention

## Abstract

From the evolutionary perspective, maintaining a committed relationship is beneficial for reproductive success but involves risks such as losing a partner or infidelity. People typically prefer partners with similar mate value (MV) to avoid rejection. However, when a mate value discrepancy (MVD) arises, the partner with lower MV might employ mate retention strategies to maintain the relationship. This study investigated whether men with lower MV compared to their female partners used cunnilingus more often and whether this effect was mediated by their motivation to satisfy the partner. Additionally, it tested the moderating role of men’s perceived vulnerability to disease (PVD), predicting that men less concerned about disease would show a stronger link between MVD and cunnilingus frequency, given the health risks associated with oral sex. Data from 540 men in committed heterosexual relationships confirmed that a higher MVD—where the man's MV was lower than his partner's—led to more frequent cunnilingus, and this relationship was mediated by a greater motivation to sexually satisfy the partner. However, the moderating role of PVD was not confirmed. We explore the evolutionary perspective that men may perform oral sex on their partners as a mate retention strategy. This behavior potentially serves as a benefit-provisioning mechanism, compensating for discrepancies in mate value.

**Supplementary Information:**

The online version contains supplementary material available at 10.1007/s10508-024-03064-4.

## Introduction

Long-term mating persists as a prevailing mode of interpersonal union across diverse human cultures (Jankowiak & Fisher, 1992). The pursuit of a committed relationship can be a driving force behind many human behaviors, decision-making processes, and strategies. From the evolutionary perspective, forming stable pair bonds ensured mutual investment in raising children, offering protection and resources essential for their well-being (Fletcher et al., 2015; Quinlan, 2008). While long-term relationships have been crucial for reproductive success and the survival of offspring, maintaining a satisfying committed relationship has been a significant adaptive challenge throughout human evolutionary history (Conroy-Beam et al., 2015, 2016).

This challenge arises from the associated fitness costs. Firstly, committing to a single mate involves a significant opportunity cost, as it limits other potentially beneficial mating opportunities for both men and women (Hurtado & Hill, 1992). In the case of men, it can reduce the overall reproductive success, especially in environments where additional mating opportunities could lead to more offspring (Hurtado & Hill, 1992; Hurtado et al., 1992). In the case of women, it may limit the possibility of securing the best genes for their offspring (Fisher, 1930; Kirkpatrick, 1996), as well as securing resources (Buss & Shackelford, 2008). Secondly, both sexes are also exposed to potential costs of their partner's infidelity (Buss & Duntley, 2011). For men, the possibility that a female partner engages in an extra-pair relationship heightens paternity uncertainty and the risk of investing resources, time, and effort into raising children that are not genetically theirs (Platek & Shackelford, 2006). For women, a partner's infidelity can mean a diversion of resources to other women. This resource dilution can compromise the support and resources available to her and her offspring, directly impacting their survival and well-being (Buss et al., 1992). Given the many potential costs associated with losing a partner, humans have evolved a range of strategies to maintain their partner's commitment, as indicated by extensive research (e.g., Apostolou et al., 2022; Lewis et al., 2013; Miner et al., 2009; Shackelford et al., 2005).

One of the strategies involves selecting a partner with a similar mate value (MV) (Buss & Shackelford, 1997), which is the overall assessment of an individual’s desirability as a romantic or sexual partner (Conroy-Beam, 2017; Csajbók et al., 2023; Edlund & Sagarin, 2014). Individuals tend to prefer partners with similar MV to avoid rejection and foster commitment in potential relationships (Tadinac & Hromatko, 2007). However, when partners' MVs begin to diverge, this mate value discrepancy (MVD) can motivate the lower MV partner to maintain the relationship (Sela et al., 2017), while the higher MV partner may be inclined to seek a better match (Conroy-Beam et al., 2016). Consequently, individuals with lower relative MV (compared to their partner) are more likely to adopt mate retention strategies, such as complimenting their partner or buying gifts (Sela et al., 2017). This behavior stems from their belief that their partners are more likely to cheat (Buss & Shackelford, 1997) and their experience of heightened jealousy (Sidelinger & Booth-Butterfield, 2007).

People employ various mate retention behaviors to minimize the risk of infidelity in their committed partners (Buss, 1988; Buss & Shackelford, 1997). According to the previous research, such behaviors may consist of cost-inflicting and benefit-provisioning strategies (Buss, 1988; Miner et al., 2009). Benefit-provisioning as opposed to cost-inflicting behaviors aim to reduce the risk of infidelity by enhancing overall relationship satisfaction, and engagement in these behaviors correlates with greater satisfaction and a lower likelihood of partner infidelity in committed relationships (Buss, 1988). These strategies may include actions dedicated to the partner's pleasure, such as giving gifts, offering compliments, or showing affection (Kaestle & Halpern, 2007; Santtila et al., 2007; Sela et al., 2017). Are these behaviors also observed in partners’ sexual activity? Indeed. For instance, oral sex has been identified as an effective strategy for mate retention (Pham & Shackelford, 2013; Sela et al., 2014). Specifically, men who reported engaging in more benefit-provisioning mate retention behaviors also showed greater interest in and spent more time performing cunnilingus—oral sex—on their partner (Pham & Shackelford, 2013). This tendency was particularly pronounced among men who perceived a higher risk of their partner's infidelity. Interestingly, these effects were not found for women (Pham et al., 2013). In addition, men typically do not perform cunnilingus on women during casual sexual encounters where there is no perceived threat of partner infidelity (Armstrong et al., 2009; Backstrom et al., 2012; Lewis et al., 2013; Reiber & Garcia, 2010).

Oral sex (particularly when received) is associated with both relationship satisfaction and sexual satisfaction (Ashdown et al., 2011; Brody & Costa, 2009; Santtila et al., 2007). Research indicates that while both men and women desire to engage in oral sex, men generally express a higher desire for it (Liu et al., 2019; Wood et al., 2016). It is noteworthy that men's relationship satisfaction positively correlates with the frequency of performing oral sex, whereas no such correlation is observed among women (Santtila et al., 2007). Importantly, women who receive oral sex from their male partners report higher levels of relationship and sexual satisfaction compared to those who do not (Kaestle & Halpern, 2007; Liu et al., 2019; Santtila et al., 2007). For instance, as has been demonstrated in Liu et al.’s study (2019), men’s giving of oral sex (and thus their female partner’s receiving of oral sex) was positively related to their own well-being through increasing their female partner’s perceived relationship quality. Therefore, if receiving oral sex is a significant factor contributing to women’s relationship satisfaction, we can hypothesize that men who are motivated to provide pleasure to their partners are more likely to perform cunnilingus. We predict that this motivation will be more pronounced among men whose mate value is lower than that of their female partner.

In summary, this study aimed to investigate whether, among heterosexual men, having a lower mate value compared to a partner's mate value leads to a higher frequency of performing active oral sex. We predicted that the greater the mate value discrepancy in favor of the female partner, the higher the man's motivation to sexually satisfy her, which would, in turn, translate into a higher frequency of performing cunnilingus. Thus, we tested whether the association between mate value discrepancy and cunnilingus frequency is mediated by the motivation to sexually satisfy the partner.

Furthermore, we included a potential moderator of the predicted effects. Although oral sex is a common sexual practice (e.g., Wood, 2016), some men simply do not engage in it. Studies indicate that the primary reason men avoid or do not enjoy cunnilingus is the perception that it is “gross” (Hattie et al., 2023). This feeling of disgust can function as a pathogen-avoidance mechanism (Curtis et al., 2011) and is a key aspect of the behavioral immune system, which evolved to protect us from infections (Ackerman et al., 2018; Murray & Schaller, 2016). Active engagement in oral sex poses a substantial risk of exposure to health-threatening pathogens. For instance, sexually transmitted infections (STIs) such as syphilis (CDC, 2004), gonorrhea, chlamydia, HPV (Edwards & Carne, 1998), and herpes (Jin et al., 2006) can be transmitted during oral sex. As the theory of the behavioral immune system states, avoidant behaviors are highly flexible and so should be performed by those who are more vulnerable to diseases (Ackerman et al., 2018). Indeed, various studies demonstrate that subjective perception of vulnerability to diseases (PVD; Duncan et al., 2009) is a significant moderator of pathogen-avoidant behaviors (Faulkner et al., 2004; Park et al., 2003, 2007; Szymkow et al., 2021). Thus, in our study, we predicted that it can significantly moderate the frequency of active oral sex. Specifically, the use of oral sex as a mate retention behavior may be more prevalent among men with lower vulnerability to disease, as they prioritize maintaining committed relationships over concerns about pathogen avoidance and health protection (Tybur et al., 2020).

Additionally, we collected data on participants' enjoyment of performing oral and vaginal sex, as well as their estimates of their partners' enjoyment of these activities. This allows us to examine potential correlations between MVD, men's enjoyment of cunnilingus and vaginal sex, women's perceived enjoyment of receiving these acts, and cunnilingus frequency. We were also able to examine whether men with lover relative MV more frequently perform oral sex on their partners, even if they do not particularly enjoy it.

## Method

The study was pre-registered and the registration is available at https://osf.io/r3c8f. All study materials are available in the Set of Questionnaires in the Supplementary Materials and in the open repository at https://osf.io/ujpwv/?view_only=d29cf1cd0d594fc1b740bfdc7bb3a0f5.

### Participants

Male university students and social media users were recruited through the SONA system (participant pool management system for universities) and Facebook announcements to take part in an online study. Student participants who took part in the study via SONA were compensated with nominal extra credit in an undergraduate course for their scientific and social activity. To qualify for participation, men had to be at least 18 years old and be currently involved in a committed, sexually active, heterosexual relationship lasting for at least 3 months. Based on these inclusion criteria, the final sample consisted of 540 men. A power analysis using G*Power 3.1.9.7 software (Faul et al., 2007) indicated that this sample size ensures power (1–β) greater than 0.80 for an effect size (*d*) surpassing 0.90.

### Procedure and Measures

Participants were informed that they are taking part in a study on sexual behaviors and were requested to complete a web-based survey via Qualtrics (Qualtrics, Provo, UT). First, participants reported their sex, age (years), type of sexual activity they most often engage in (exclusively heterosexual, predominantly heterosexual, bisexual, predominantly homosexual or exclusively homosexual, or not sexually active), and current relationship length (less than 3 months, between 3 and 6 months, between 6 and 12 months, more than 1 year, or more than 3 years). Individuals who did not meet the inclusion criteria were excluded from the survey at this stage. Following that, participants completed a series of scales and measurements. The main dependent variable in the study was the frequency of performing cunnilingus on a participant's female partner. Participants responded to questions about how many of their last 10 sexual encounters involved orally stimulating their committed partner (regardless of whether it was part of foreplay or a replacement for vaginal intercourse). The predictor variables in the study included mate value discrepancy, motivation to sexually satisfy the partner, and subjective perceived vulnerability to disease.

#### Mate Value Discrepancy

The mate value discrepancy between participants and their committed partners was measured using the Mate Value Scale (Edlund & Sagarin, 2014). Participants used the scale to assess their own MV and separately evaluate the perceived MV of their committed partners. The scale consisted of four questions about perceived MV, reflecting how individuals view themselves compared to others in the dating market. Specifically, participants read the following instructions: "Many people pay attention to specific traits when choosing a potential romantic partner. Some commonly desired traits include: being socially engaging, age, physical attractiveness, having a sense of humor, being kind and understanding, having high financial/professional status, high intelligence, good health, and whether or not one likes children. Those who possess these traits to a high degree are highly sought after by the opposite sex and are said to have a high mate value. With this in mind, we would like you to try to assess both your own mate value and separately the mate value of your romantic partner in the order indicated on the following pages.” After reading this, participants were asked to rate themselves and their partners on a 7-point scale (from 1 = *extremely low* to 7 = *extremely high*) in response to the following four questions: “Overall, how would you rate your level of desirability as a partner?” “Overall, how would members of the opposite sex rate your level of desirability as a partner?” “Overall, how do you believe you compare to other people in desirability as a partner?” “Overall, how good of a catch are you?” The MVD was calculated by subtracting the average MV of the participant (Cronbach’s *α* = .88) from the average MV of their partner (Cronbach’s *α* = .86). Thus, the higher the MVD value, the higher the MV of a woman in comparison with a man’s MV.

#### Motivation to Sexually Satisfy the Committed Partner

To gauge participants’ general motivation to sexually satisfy their committed partner, we developed four items specifically for this purpose. Participants rated their agreement with each statement on a 7-point scale (from 1 = *strongly disagree* to 7 = *strongly agree*). Statements included: “It is most important for me to sexually satisfy my partner,” “During sexual intercourse, I engage in additional activities that I know are particularly enjoyable for my partner,” “During intimacy, I primarily focus on my own pleasure” (this item has been recoded), and “I prioritize my partner's sexual satisfaction over mine during sexual intercourse.” The general motivation to sexually satisfy the committed partner was calculated by averaging answers to these four questions (Cronbach’s α = .62).

#### Perceived Vulnerability to Disease

Subjective perceptions of susceptibility to disease were measured using the Perceived Vulnerability to Disease Scale (PVD; Duncan et al., 2009). The scale comprised 15 statements (e.g., "I prefer to wash my hands shortly after shaking someone's hand") assessed on a 7-point scale (from 1 = *strongly disagree* to 7 = *strongly agree*). The scale included two subscales: Perceived Infectability (PI; Cronbach’s α = 0.82) and Germ Aversion (GA; Cronbach’s α = 0.69). The items for each subscale were averaged to create two indices, which were analyzed separately.

#### Additional Measures

We also measured the perceived extent of enjoyment felt by their committed partners when being orally and vaginally pleased by them, as well as the extent of their own enjoyment of pleasing their partners orally or vaginally. Statements included: “How much do you think your partner enjoys being satisfied by you through oral sex?” “How much do you enjoy satisfying your partner orally?” “How much do you think your partner enjoys being satisfied by you through vaginal sex?” and “How much do you enjoy satisfying your partner through vaginal sex?” Participants rated their agreement with each statement on a 7-point scale (from 1 = *She/I strongly dislike(s) it* to 7 = *She/I strongly like(s) it*).

### Statistical Analyses

According to our pre-registered plan of analyses, we first calculated descriptive statistics and simple Pearson correlation analyses to test linear correlations between MVD, the frequency of engaging in active oral sex, and motivation to sexually satisfy the female romantic partner, using IBM SPSS Statistics 29. In the next step, we used SPSS Process, macro model 4 (see Hayes & Rockwood, 2017), to test whether the relationship between MVD and the frequency of engaging in active oral sex is mediated by the motivation to sexually satisfy the female romantic partner. Finally, we used model 7 (see Hayes & Rockwood, 2017) to test whether this mediation is moderated by the participant's perceived vulnerability to disease. As recommended by Hayes (2013), the regression/path coefficients were unstandardized. We have also run additional non-pre-registered exploratory analyses which are presented in the last section of results.

## Results

### Characteristics of Participants

Participants were between 18 and 80 years of age (*M* = 29.50, *SD* = 11.58) and were in committed, sexually active, heterosexual relationships lasting for at least 3 months. The final sample consisted of 540 men: 518 (95.9%) engaging in exclusively heterosexual sexual activities, and 22 (4.1%) engaging in predominantly heterosexual activities. Fifty-four (10%) were in relationships lasting between 3 and 6 months, 42 (7.8%) between 6 and 12 months, 112 (20.7%) more than 1 year, and 332 (61.5%) more than 3 years.

### Initial Analyses

Descriptive statistics and correlation coefficients between continuous variables are presented in Table 1. We found that MVD correlated positively with the motivation to sexually satisfy one’s female partner (*r = .*13, *p = .*002), which means that as the mate value discrepancy in favor of the female partner increases, so does the motivation of men to sexually satisfy their partners. However, there was no zero-order correlation between MVD and the frequency of performing cunnilingus (*r = .*04, *p = .*295). The motivation to sexually satisfy the partner was positively correlated with the frequency of performing cunnilingus (*r = .*27, *p < .*001), which means that the more motivated a man was to sexually satisfy his female partner, the more frequently he performed active oral sex.

**Table 1:** Zero-order correlations among independent and dependent variables

Variables	*M*	*SD*	1	2	3
1. Mate value discrepancy (MVD)	.63	1.09			
2. Motivation to satisfy the partner	5.59	.92	.13**	–	
3. Cunnilingus frequency	5.02	3.33	.04	.27***	–

Cell entries are zero-order Pearson correlation coefficients (two-tailed), *p < .05, **p < .005, and ***p < .001. MVD = subtracted mate value of a woman from the mate value of a man

### The Mediating Role of Motivation to Sexually Satisfy the Partner

**Fig. 1:**
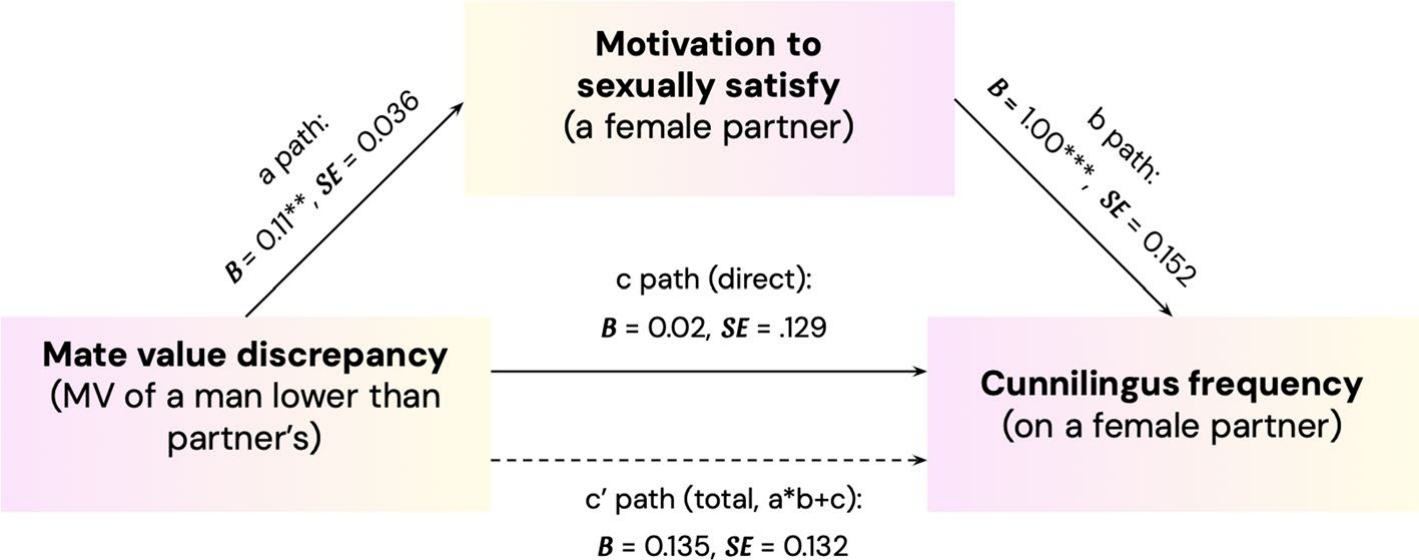
Indirect effect of mate value discrepancy on cunnilingus frequency through motivation to sexually satisfy a female partner. *Note* *p < .05, **p < .005, and ***p < .001. The model included two covariates: participant age and relationship length

To test whether the relationship between MVD and the frequency of engaging in active oral sex is mediated by the motivation to sexually satisfy the female romantic partner, we used SPSS Process, macro model 4 (see Hayes & Rockwood, 2017). We present the mediation diagram in Fig. 1. Since participants' age and relationship length may affect the frequency of oral sex, we added these variables as covariates in the analysis. The results of the mediation analysis (Mate value discrepancy → Motivation to satisfy partner → Cunnilingus frequency) revealed that the predicted indirect effect of the mediation pattern was significant, as indicated by the fact that the 95% CI did not include zero, *b* = 0.110, 95% CI = [0.029, 0.205]. This finding is consistent with the hypothesis that the higher the discrepancy between mate value of a man and his female partner in favor of the partner, the higher his motivation to satisfy his partner, path a: *b* = 0.110, 95% CI = [0.039, 0.182], which further predicts his active oral sex frequency, and path b: *b* = 0.999, 95% CI = [0.700, 1.298]. Importantly, the direct effect of the MVD on active oral sex frequency was not significant, *b* = 0.025, 95% CI = [− 0.228, 0.277], which signals the full mediation model. It means that mate value discrepancy does not influence cunnilingus frequency directly, but indirectly, by affecting the man’s motivation to satisfy his partner, which further translates into active oral sex frequency. All specific coefficients for direct and indirect paths are presented in Table 2.

**Table 2:** Coefficients of direct effects of mediation analysis of the relationship between mate value discrepancy and frequency of performing cunnilingus with one mediator: motivation to satisfy the partner

	Motivation to satisfy the partner (M)						Cunnilingus frequency (Y)						Indirect effect coefficients			
	*b*	*SE*	*t*	*p*	*CI* 95%*		*b*	*SE*	*t*	*p*	*CI* 95%*		*b*	*SE*	*CI* 95%*	
					*LL*	*UL*					*LL*	*UL*			*LL*	*UL*
*Direct effects paths*																
Mate value discrepancy (X)	0.11	0.036	3.041	.003	0.039	0.182	0.025	0.129	0.191	.849	− 0.228	0.277				
Motivation to satisfy the partner (M)							0.999	0.152	6.561	.000	0.700	1.298				
Total effect X → Y							0.135	0.132	1.018	.309	− 0.125	0.395				
*Indirect effect*																
MVD → Motivation to satisfy the partner → Cunnilingus frequency													0.110	0.045	0.029	0.205

Covariates: participant age, relationship length

*95% CI is presented as bias-corrected and accelerated 5000 bootstrapping

*MVD* mate value discrepancy

### The Moderating Role of Perceived Vulnerability to Disease

First, we calculated zero-order correlations between PVD indices and active oral sex frequency. Both germ aversion (*r = − .*07, *p = .*043, one-tailed) and perceived infectability (*r = − .*09, *p = .*032) were negatively, but very weakly, related to active oral sex performance. It means that the more the participants perceived themselves as vulnerable to disease, the less likely they were to perform oral sex on their female partner. To test whether the indirect relationship between MVD and the frequency of engaging in active oral sex is moderated by the perceived vulnerability to disease (PVD), we used SPSS Process, macro model 7 (see Hayes & Rockwood, 2017). Along with our pre-registered plan of analyses, we run two separate moderated mediation analyses: one for germ aversion (GA) as a moderator and one for perceived infectability (PI) as a moderator (Duncan et al., 2009).

### Germ Aversion as a Moderator

Moderated mediation analysis for the frequency of active oral sex as the outcome variable, MVD as a predictor, motivation to satisfy the female partner as a mediator, GA as a moderating variable, and participant age and relationship length as covariates, revealed that the moderation effect was not significant, *b* = 0.020, 95% CI = [− 0.057, 0.104]. It means that the mediating effect of motivation to satisfy a partner in a relationship between mate value discrepancy and active oral sex frequency held true independently of germ aversion levels. Detailed results are presented in Supplementary Table 1.

### Perceived Infectability as a Moderator

Moderated mediation analysis for the frequency of active oral sex as the outcome variable, MVD as a predictor, motivation to satisfy the female partner as a mediator, PI as a moderating variable, and participant age and relationship length as covariates, revealed that the moderation effect was not significant, *b* = 0.018, 95% CI = [− 0.065, 0.093]. It means that the mediating effect of motivation to satisfy a partner in a relationship between mate value discrepancy and active oral sex frequency held true independently of perceived infectability levels. Detailed results are presented in Supplementary Table 2.

### Additional Exploratory Analyses

As we included some additional measures in our study, namely, the extent to which men declared liking to perform active oral and vaginal sex on their female partners, as well as the extent to which they estimated their partners’ enjoyment of receiving oral and performing vaginal sex, we decided to run extra exploratory analyses which were not pre-registered.

First, we calculated zero-order correlations between MVD, enjoyment of performing cunnilingus and vaginal sex by men, perceived enjoyment of receiving cunnilingus and vaginal sex by their female partners, and men’s cunnilingus frequency. There was a substantial correlation between the extent to which men believed their female partners enjoyed being satisfied orally and the frequency of cunnilingus performed (*r = .*61, *p < .*001). Also, the extent to which men enjoyed orally satisfying their female partners correlated significantly with the frequency of active oral sex (*r = .*46, *p < .*001). Interestingly, the index of mate value discrepancy was positively correlated with men’s enjoyment of orally satisfying their female partners (*r = .*12, *p = .*006). This means that the higher the discrepancy between a man's mate value compared to his female partner's mate value in favor of a woman, the more likely he was to enjoy performing cunnilingus. Detailed results are presented in Supplementary Table 3.

In the next step, we investigated whether the established mediational effect (Mate value discrepancy → Motivation to satisfy the partner → Cunnilingus frequency) depends on men’s enjoyment of performing cunnilingus on their female partners. To do that, we used SPSS Process, macro model 14 (see Hayes & Rockwood, 2017), introducing men’s enjoyment of active oral sex as a moderator variable, and participant sex and relationship length as covariates. The analysis revealed that the moderated mediation index was significant, *b* = 0.015, 95% CI = [0.002, 0.033]. The mate value discrepancy translated into higher frequency of active oral sex via the motivation to satisfy one’s partner at the moderate and high values of active oral sex enjoyment, but the effect was absent at the low level of enjoying active oral sex: the coefficient on 1 *SD* below the mean was *b* = 0.168; 95% CI = [− 0.167, 0.504], on the mean was *b* = 0.374; 95% CI = [0.061, 0.687], and on 1 *SD* above the mean was *b* = 0.519; 95% CI = [0.146, 0.891]. Those men who did not like to perform oral sex on their female partners were not inclined to do that despite experiencing high mate value discrepancy. Apparently, in their case, active oral sex does not serve as a compensatory strategy. Detailed results are presented in Supplementary Table 4.

## Discussion

The results support the hypothesis that men who perceive a greater MVD favoring their female partners are more motivated to sexually satisfy them, resulting in more frequent cunnilingus. This aligns with research suggesting that MVD encourages low mate value partners to engage in mate retention behaviors, aiming to maintain long-term relationships and reduce the risk of losing a partner or facing infidelity (Miner et al., 2009; Pham & Shackelford, 2013). Additionally, men in long-term, committed relationships, who declare loving their partners “a lot,” are also more likely to perform oral sex on their partners (Kaestle & Halpern, 2007). In fact, cunnilingus is an effective tool in providing sexual satisfaction to a female partner. Women who receive oral sex are more likely to experience orgasm during a sexual encounter compared to those who do not (Backstrom et al., 2012). Furthermore, men who induce their partner's orgasm are perceived as significantly more masculine, particularly those with low testosterone levels (Hawley et al., 2024). Consequently, men might engage in cunnilingus to enhance their value in the eyes of their partner, thereby reducing the mate value discrepancy. Future research should directly investigate whether oral sex performed by men with lower mate value than their partner's actually results in female orgasm. Our study did not explore this aspect, so it remains unclear how many instances of cunnilingus led to orgasm.

As evolutionary mechanisms are by no means deterministic—on the contrary, they are highly conditional (Krebs & Davies, 2009), and dependent on factors related to specific situational contexts, as well as to individual characteristics of actors involved (Buss & Greiling, 1999), we expected that using cunnilingus as a MVD compensatory strategy would not be universal. Specifically, we predicted that men expressing high levels of perceived vulnerability to disease (i.e., germ aversion and perceived infectability) would not show the predicted pattern of results. However, we found no moderation effect. Men with lower MV than their partners and higher PVD did not engage in oral sex less frequently to satisfy their partners compared to those with lower PVD. This was surprising, as according to behavioral immune system theory (Faulkner et al., 2004; Park et al., 2003), individuals with higher levels of perceived vulnerability to disease are typically more sensitive to disgust and tend to avoid behaviors that pose a heightened pathogen threat. The lack of support may be due to the high generality of the PVD scale used. We measured PVD using the Perceived Vulnerability to Disease Scale (Duncan et al., 2009), which includes perceived infectability and germ aversion but does not directly measure disgust. Given the multifaceted nature of disgust, with its various aspects (such as pathogen, moral, and sexual disgust) addressing different functional problems (Tybur et al., 2009), it may be more appropriate to use a specific measure like sexual disgust to capture the precise reaction people have toward oral sex. Additionally, there is a clear trade-off between two motives: mate retention and pathogen avoidance. In terms of reproductive success, prioritizing mate retention may be more beneficial than avoiding pathogens. However, our findings do not fully support this logic. Specifically, our results show that men who do not enjoy performing cunnilingus are not inclined to do so, despite experiencing mate value discrepancy. For these men, performing oral sex does not appear to serve as a compensatory strategy for MVD. Future studies should explore these issues further.

It must be noted that our participants were only men, but similar predictions likely apply to women. Specifically, women who report higher MVD favoring their male partners should be more motivated to engage in mate retention behaviors, such as sexually satisfying their partners, including through oral sex. Supporting this, Sela et al. (2014) found that women who report performing more benefit-provisioning mate retention behaviors also report greater interest in and spend more time performing oral sex on their partners. This suggests that both men and women perform oral sex as part of a benefit-provisioning strategy to increase their partner’s relationship satisfaction. Future studies should focus on direct comparisons between men and women.

There are limitations to the current study. First, we relied on men’s self-reports of their frequency of performing oral sex and their motivation to sexually satisfy their partners. Men may overreport socially desirable behaviors, such as caring for their partner's sexual satisfaction. However, the previous research has shown that self-reports of mate retention behaviors positively correlate with partner reports (Shackelford et al., 2005). Moreover, these declarations were predicted by MVD and were present only among men who enjoy oral sex, suggesting that they do not need to overestimate such activities as they also derive pleasure from them. It is also possible that men reported these behaviors to heighten their own MV, as being good at sex is a crucial part of MV (e.g., Back et al., 2011). If this was the case, it would also apply to men who do not enjoy oral sex, as they too should be motivated to increase their MV. Nonetheless, future research may benefit from securing data from both self-reports and partner reports of men’s actual sexual activity and their partners' actual sexual satisfaction.

Another limitation is that we do not address cultural differences in sexual activities that serve as mate retention behavior. Our data were gathered in Poland, a country with an individualistic culture where oral sex is not generally considered taboo and is becoming increasingly common (Izdebski, 2020). However, while oral sex is a common sexual activity practiced across many cultures (e.g., Guadamuz et al., 2010; Santtila et al., 2007), it is considered strange and unusual in several other cultures and religions (Keating, 1988; Pakpahan et al., 2022). It is also important to note that Poland's relatively low pathogen burden might influence perceived vulnerability to disease and disgust sensitivity (Miłkowska et al., 2021; Szymkow et al., 2021; Tybur et al., 2016), potentially affecting oral sex practices. Future research should explore differences between populations from countries with diverse pathogen burdens and cross-cultural differences when investigating oral sex as a mate retention strategy.

Aside from the limitations mentioned above, several other questions have emerged from this study, highlighting the need for further research. Participants were asked how many of their last 10 sexual encounters included orally stimulating their partner. Since this was the only information collected about these encounters, it leaves significant aspects unexplored. For example, without data on the overall frequency of sexual activity, variations in cunnilingus frequency might partly reflect low sexual activity, with cunnilingus serving as a strategy to increase intercourse frequency. Additionally, examining who typically initiates sexual encounters and how often men’s sexual advances are rejected by their partners could provide valuable insights. These dynamics might clarify how motivation for cunnilingus develops and how such factors influence perceived MVD. For instance, a man who frequently experiences sexual rejection from his partner over time may begin to perceive an MVD that was not evident at the start of the relationship. Alternatively, the time since the last sexual encounter might indicate a period of abstinence, which could independently—or perhaps even more strongly—drive sexual motivation across various activities, including cunnilingus. These questions merit further exploration in the future research.

Our results suggest that performing oral sex to sexually satisfy a committed partner may serve as a dynamic mate retention tactic related to the MV of both partners, consistent with the previous studies (Miner et al., 2009). However, it is possible that preferences for performing oral sex are stable across different relationships and may not be influenced by MVD. Alternatively, oral sex might simply serve as a fixed part of a sexual encounter. Our data do not allow us to answer this fully, as we have only gathered men’s declarations. Future research could benefit from investigating individual differences in men’s oral sex behaviors and their stability across different sexual relationships. Also, even though oral sex is currently independent of age and gender (Pakpahan et al., 2022), it can still be associated with factors such as sociosexuality, religiosity, and personality traits. For instance, men who are more agreeable and altruistic might be more inclined to provide benefits to their partners, such as sexually satisfying them by performing cunnilingus. So far, data linking engaging in oral sex with personality traits from the Five-Factor Personality Model exist only for women. Sela and colleagues (2015) showed that women higher in conscientiousness and agreeableness report greater interest in and spend more time performing fellatio on their partners, and this relationship is mediated by their benefit-provisioning mate retention. While a similar pattern is likely for men, future research could test this by assessing and statistically controlling for altruism-linked personality traits that correlate with partner-directed mate retention behaviors.

In conclusion, from the evolutionary perspective, men experiencing lower mate value comparing to his female partner’s mate value, may employ various mate retention behaviors, including sexual activities, to prevent reproductive costs such as losing a partner or cuckoldry as the result of partner’s infidelity—the inadvertent investment of time and resources into genetically unrelated offspring. Our study indicates that low mate value men feel more motivated to sexually satisfy their female partners and that this motivation translates into cunnilingus frequency. This research, along with the previous studies (Pham & Shackelford, 2013; Sela et al., 2015), suggests that both men and women perform oral sex on their partners as a benefit-provisioning mate retention behavior. This extends to the potential mechanism of such behaviors being a form of compensation for mate value discrepancy.

## Supplementary Information

Below is the link to the electronic supplementary material.

Supplementary file1 (PDF 349 kb)

## Data Availability

Dataset and all study materials are available in the Set of Questionnaires in the Supplementary Materials and in the open repository at: https://osf.io/ujpwv/?view_only=d29cf1cd0d594fc1b740bfdc7bb3a0f5.
