# Supplementary material for: Oral Sex May Serve as Low Mate Value Compensation Among Men: Evidence from a Pre-registered Study
Source: Arch Sex Behav. 2024 Dec 26;54(3):893–903. doi: 10.1007/s10508-024-03064-4 (PMC11926041; doi:10.1007/s10508-024-03064-4)
Supplement: Supplementary file 1 — Supplementary file1 (PDF 349 kb) [file 10508_2024_3064_MOESM1_ESM.pdf]

Supplementary Materials

To accompany the manuscript "Oral sex may serve as low mate value compensation among men: Evidence from a preregistered study"

Set of Questionnaires

Mate value discrepancy (The Mate Value Scale[21])

Many people look at specific characteristics in choosing their potential marriage partners. Some common desirable traits include: being socially exciting, age, being physically attractive, having a good sense of humor, being kind and understanding, having good financial/professional status, being of high intelligence, being in good health, and liking children:

Overall, how would you rate your level of desirability as a partner on the following scale?

1	2	3	4	5	6	7
Extremely undesirable						Extremely desirable

Overall, how would members of the opposite sex rate your level of desirability as a partner on the following scale?

1	2	3	4	5	6	7
Extremely undesirable						Extremely desirable

Overall, how do you believe you compare to other people in desirability as a partner on the following scale?

1	2	3	4	5	6	7
Very much lower than average	Lower than average	Slightly lower than average	Average	Slightly higher than average	Higher than average	Very much higher than average

Overall, how good of a catch are you?

1	2	3	4	5	6	7
Very bad catch	Bad catch	Somewhat bad of a catch	Average catch	Somewhat good of a catch	Good catch	Very good catch

Motivation to sexually satisfy the committed partner

For each statement about sexual encounters with your committed partner, please check the box that best represents your level of agreement on a scale from 1 = strongly disagree to 7 = strongly agree:

1. It is most important for me to sexually satisfy my partner.
2. During a sexual encounter, I engage in additional activities that I know are particularly enjoyable for my partner.
3. During intimacy, I primarily focus on my own pleasure. R
4. I prioritize my partner's sexual satisfaction over mine during a sexual encounter.

Perceived vulnerability to disease (PVD scale[45])

For each statement considering your thoughts about how diseases are spreading - please check the box corresponding to the answer that best represents your level of agreement with each of them on a scale from 1 = strongly disagree to 7 = strongly agree:

1. It really bothers me when people sneeze without covering their mouths.
2. If an illness is 'going around', I will get it.
3. I am comfortable sharing a water bottle with a friend. R
4. I don't like to write with a pencil someone else has obviously chewed on.
5. My past experiences make me believe I am not likely to get sick even when my friends are sick. R
6. I have a history of susceptibility to infectious diseases.
7. I prefer to wash my hands pretty soon after shaking someone's hand.
8. In general, I am very susceptible to colds, flu, and other infectious diseases.

9. I dislike wearing used clothes because you don't know what the past person who wore it was like.
10. I am more likely than the people around me to catch an infectious disease.
11. My hands do not feel dirty after touching money. R
12. I am unlikely to catch a cold, flu, or other illness, even if it is going around. R
13. It does not make me anxious to be around sick people. R
14. My immune system protects me from most illnesses that other people get. R
15. I avoid using public telephones because of the risk that I may catch something from the previous user.

Tables

Supplementary Table 1

Moderated Mediation Model 7: Indirect effect of Mate Value Discrepancy (IV) on Cunnilingus Frequency (DV) through Motivation to Satisfy the Partner (M) moderated by Germ Aversion (Mo), Covariates: participant age, relationship length, N = 536.

	**Mediator variable model (DV = Motivation)**			
**Predictors**	*b*	SE	t-Value	p-Value
Mate value discrepancy (MVD)	0.036	0.127	0.286	.775
Germ aversion (GA)	-0.033	0.044	-0.743	.458
MVD x GA	0.020	0.034	0.602	.548
	**Dependent variable model (DV = Cunnilingus Frequency)**			
**Predictors**	*b*	*SE*	*t*-Value	*p*-Value
Mate value discrepancy (MVD)	0.025	0.129	0.191	.849
Motivation to satisfy the partner	0.999	0.152	6.561	.000
	**Conditional Indirect Effect at GA +/- 1 SD**			
**Moderator**	Moderator value	*b*	Boot *SE*	Boot 95% CI*
	(-1 SD) 2.68	0.090	0.052	[-.01, .19]
Germ Aversion (GA)	(Mean) 3.70	0.111	0.044	[.03, .21]
	(+1 SD) 4.72	0.131	0.068	[.01, .28]
**Index of moderated mediation**		0.020	0.040	[-.06, .10]

*Note. * 95% CI is presented as bias-corrected and accelerated 5,000 bootstrapping.*

Supplementary Table 2

Moderated Mediation Model 7: Indirect effect of Mate Value Discrepancy (IV) on Cunnilingus Frequency (DV) through Motivation to Satisfy the Partner (M) moderated by Perceived Infectability (Mo), Covariates: participant age, relationship length, N = 536.

	**Mediator variable model (DV = Motivation)**			
**Predictors**	*b*	SE	t-Value	p-Value
Mate value discrepancy (MVD)	0.050	0.107	0.465	.642
Perceived Infectability (PI)	-0.096	0.040	-2.419	.016
MVD x PI	0.018	0.028	0.632	.527
	**Dependent variable model (DV = Cunnilingus Frequency)**			
**Predictors**	*b*	*SE*	*t*-Value	*p*-Value
Mate value discrepancy (MVD)	0.025	0.129	0.191	.849
Motivation to satisfy the partner	0.999	0.152	6.561	.000
	**Conditional Indirect Effect at GA +/- 1 SD**			
**Moderator**	Moderator value	*b*	Boot *SE*	Boot 95% CI*
	(-1 SD) 2.41	0.093	0.053	[.002, .21]
Perceived Infectability (PI)	(Mean) 3.54	0.113	0.042	[.04, .20]
	(+1 SD) 4.66	0.133	0.079	[.002, .27]
**Index of moderated mediation**		0.018	0.040	[-.07, .09]

Note. * 95% CI is presented as bias-corrected and accelerated 5,000 bootstrapping.

Supplementary Table 3

Zero-order correlations among independent and dependent variables

**Variables**	1	2	3	4	5	6
1. Mate value discrepancy (MVD)	-					
2. Men's liking of performing cunnilingus	.12**	-				
3. Men's liking of performing vaginal sex	.06	.31***	-			
4. Women's liking of receiving cunnilingus (perceived by men)	.00	.33***	.17***	-		
5. Women's liking of performing vaginal sex (perceived by men)	.11*	.14***	.43***	.17***	-	
6. Cunnilingus frequency	.04	.46***	.14***	.61***	.04	-

Note. Cell entries are zero-order Pearson correlation coefficients (two-tailed), **p* < .05, ***p* < .01, ***p < .001. MVD = subtracted the mate value of a man from the mate value of his female partner.

Supplementary Table 4

Moderated Mediation Model 14: Indirect effect of Mate Value Discrepancy (IV) on Cunnilingus Frequency (DV) through Motivation to Satisfy the Partner (M) moderated by Men's liking of performing cunnilingus on their female partner (Mo), Covariates: participant age, relationship length, N = 536.

	**Mediator variable model (DV = Motivation)**			
**Predictors**	*b*	SE	t-Value	p-Value
Mate value discrepancy (MVD)	0.110	0.036	3.041	.003
	**Dependent variable model (DV = Cunnilingus Frequency)**			
**Predictors**	*b*	*SE*	*t*-Value	*p*-Value
Mate value discrepancy (MVD)	-0.035	0.118	-0.299	.765
Motivation to satisfy the partner	-0.437	0.411	-1.063	.288
Liking of performing cunnilingus	0.263	0.366	0.718	.473
Motivation x Liking	0.137	0.070	1.954	.051
	**Conditional Indirect Effect at GA +/- 1 SD**			
**Moderator**	Moderator value	*b*	Boot *SE*	Boot 95% CI*
	(-1 SD) 4.43	0.019	0.019	[-.01, .06]
Liking of performing cunnilingus	(Mean) 5.94	0.041	0.024	[.002, .10]
	(+1 SD) 7.00	0.057	0.030	[.007, .13]
**Index of moderated mediation**		0.015	0.008	[.002, .03]

*Note. * 95% CI is presented as bias-corrected and accelerated 5,000 bootstrapping.*
